# The (un)controlled body: A grounded theory analysis to conceptualise stigma for women with gestational diabetes mellitus

**DOI:** 10.1177/13591053241241863

**Published:** 2024-04-16

**Authors:** Madeleine Benton, Natasha Hotung, Jessica Bird, Khalida Ismail, Sergio A Silverio

**Affiliations:** 1 King’s College London, UK; 2 Liverpool John Moores University, UK

**Keywords:** maternal health, perinatal health, pregnancy

## Abstract

Health-related stigma is associated with adverse outcomes including depression, stress and reduced engagement in health behaviours which are particularly harmful in pregnancy and the postpartum. Women with gestational diabetes mellitus (GDM) report negative psychosocial experiences and may be at risk of stigma related to the condition. We aimed to understand women’s experiences of GDM-specific stigma. Individual interviews were conducted with *n* = 53 women living in the UK with a current or past (within 4 years) GDM. Grounded theory methodology was used to analyse the data. Four themes were identified: (1) Preconceptions and misconceptions; (2) Locating, regaining, and negotiating agency; (3) Tension about and resisting the dominant discourse of stigma; and (4) Reclaiming control over the body. GDM-specific stigma was diverse and far reaching and may have broader implications for perinatal mental health and postnatal wellbeing. It is pertinent to investigate possible prospective associations between GDM-specific stigma, and biomedical and mental health outcomes.

## Background

Gestational diabetes mellitus (GDM) is a form of glucose intolerance resulting in an excess of sugar in the blood (hyperglycaemia), which develops in pregnancy and resolves after birth (World Health Organization, 1999). It is the most common pregnancy complication, estimated to affect approximately 15% of pregnancies worldwide (Wang et al., 2022). The potential adverse outcomes related to GDM for women and their babies have been consistently reported (Ye et al., 2022). Short-term adverse outcomes can include pre-eclampsia, a large-for-gestational age baby, increased need for induction of labour and caesarean section (Ye et al., 2022). While GDM usually resolves after birth, it has long-lasting health consequences, including a 50% increased risk for GDM recurrence and type 2 diabetes (T2D) in the mother and future obesity, as well as T2D and neurodevelopmental problems in the child (Bellamy et al., 2009; Vounzoulaki et al., 2020).

The management of GDM aims to optimise maternal blood glucose levels using first line intensive lifestyle modification (to diet and exercise) followed second line with insulin therapies (often in the form of injections) and/or (tablet form) metformin (American Diabetes Association, 2021). As part of GDM management, women are also required to monitor their blood glucose, and attend additional medical appointments. Consequently, GDM pregnancies become highly medicalised (Diane et al., 2017).

GDM is recognised to potentially induce a range of negative emotional responses, such as feelings of shame, guilt and fear, and a sense of deviation from the normative pregnancy experience (Craig et al., 2020). Literature highlights that women often feel ‘blamed’ for their GDM diagnosis as it is assumed their condition is due to individual lifestyle choices (Davidsen et al., 2022; McNaughton, 2011). Furthermore, there is increasing evidence of the association between GDM and the development of mental health symptomatology’s, notably depression and anxiety (Wilson et al., 2020).

Stigma is a complex, multifaceted phenomenon which incorporates aspects of the personal, inter-personal relationships, and the societal hegemony. It is characterised by cognitive, emotional and behavioural components and can be reflected both in attitudes and experiences (Hatzenbuehler et al., 2013). Stigmatised attitudes are often conceptualised as perceived (how others act, think, feel towards someone based on a certain trait or identity), anticipated (the expectation of future stigmatisation), or internalised stigmas (the process of accepting and applying stigma to oneself) (Kane et al., 2019). While experienced (or enacted) stigmas involve discriminatory acts or behaviours (Scambler and Hopkins, 1986). Stigma adversely affects health outcomes; it is a barrier to health-seeking behaviour, engagement in care and adherence to treatment, across a range of health conditions (Kane et al., 2019; Stangl et al., 2019). It worsens, undermines and impedes a number of processes, including social relationships, resource availability, stress and psychological and behavioural responses, exacerbating poor health (Hatzenbuehler et al., 2013).

Weight stigma is well-studied (Wu and Berry, 2018), and there has been increasing attention paid to weight stigma among pregnant and postpartum women (Heslehurst et al., 2022; Incollingo Rodriguez et al., 2019, 2020; Mulherin et al., 2013). Weight stigma can increase depressive symptoms (Incollingo Rodriguez et al., 2019), and forms a vicious cycle, where weight stigma increase physiological stress, increasing food consumption, binge eating, cortisol production leading to weight retention, weight gain and reinforcing or increasing weight-based stigma (Puhl and Suh, 2015). Furthermore, stigma related to type 1 and type 2 diabetes in adult populations has been previously described. Research has highlighted that individuals with diabetes often encounter societal perceptions that attribute their condition to personal failures, such as lifestyle choices or character flaws (Liu et al., 2017). This stigma can manifest in negative stereotypes and discriminatory attitudes, contributing to heightened stress, shame and psychological distress for those living with diabetes. Given the increasing evidence of the detrimental effects of weight stigma during and after pregnancy and of diabetes stigma more generally, it becomes necessary to also consider the potential stigma associated with GDM. This study aimed to understand how women with GDM experience potential stigma related to the condition.

## Methods

### The present study

The study presented below was an extension of a smaller study examining the impact of GDM on women’s psychosocial outcomes including mother-infant bonding (Benton et al., 2023). This work comprised interviews with women (*n* = 33) where stigma arose as a recurring theme. In-line with Grounded Theory Analysis approaches, the team decided further investigation on the topic was needed and theoretically sampled for more participants to specifically address the impact of GDM on women’s ‘social experiences’. We purposefully did not refer to ‘stigma’ during study recruitment in order to minimise the risk of inadvertently attracting only participants with extreme negative experiences and to avoid biasing participants’ interview responses. The aim of the current study and analysis presented below was to understand women’s experiences of stigma related to GDM.

### The study team

The study was conducted by a cross-disciplinary team, with expertise in women’s health (MB, SAS), psychology (MB, NH, JB, SAS), diabetes (MB, KI) and psychiatry (KI). MB has training in qualitative research and SAS has recognised expertise in Grounded Theory, including its development and application. Grounded Theory warrants – where possible – the ideal circumstance of no *a priori* assumptions or the ‘blank slate’ *tabula rasa-*researcher (Glaser and Strauss, 1967). Given this study falls within the expertise of some of the authorship team (MB, KI) it was important to understand where any preconceived notions and/or biases were being introduced by members of the research team. Researchers therefore kept memo notes during data collection and analysis to aid bracketing (Gearing, 2004) – that is, to recognise and extricate coding or analysis which was not grounded in the data and emergent through the analytical processes. Attending to these aspects throughout the analysis and interpretive phases allowed the team to notice where their preconceptions were evident and remove them in order to maintain the desired *tabula rasa* free from *a priori* assumptions within the analytical work, and therefore maintaining the principles upon which Grounded Theory Analysis is based. This was further aided by the senior qualitative co-author (SAS) who is not an expert in GDM, and therefore could provide unbiased advice on theme and theory generation based purely on the data presented. This is in-keeping with much of the contemporary literature on the learning and ‘doing’ of Grounded Theory Analysis (McCallin, 2003; Silverio et al., 2019; Tarozzi, 2020).

### Theoretical perspective

This study was informed by gendered lifecourse analysis approaches (Wainrib, 1992) and adopted a theoretical perspective accordingly. In brief, this recognises that in Western settings (such as the UK), women’s normative lifecourses usually include pregnancy and childbirth, and the site of empirical inquiry here is the transition from pregnant woman to motherhood. A lifecourse perspective is in harmony with Grounded Theory Analysis and is acknowledged by Glaser and Strauss (1967) who note that researchers should be aware of how lives are demarcated by transition points. We were therefore seated within a post-positivist research paradigm (Annells, 1996; Levers, 2013), underpinned philosophically by a critical realist ontology and an objectivist epistemology; and engaging in principles of positionality including critical reflexivity and an objective outsider position in relation to the data. Critical realism interprets lifecourses through a lens which recognises social contexts and social conditioning (Roberts, 2014) and therefore acknowledges the lived truth of people’s realities, even if the recounting of said events is not necessarily true. Epistemological objectivism assumes researchers will adopt objectivist principles, refuting the construction of reality and arguing that a reality exists (Greckhamer and Koro-Ljungberg, 2005). Critical reflexivity suggests we engage with participants’ narratives in a way which understands the value-laden concepts about which they speak, but with acknowledgement that changing structural conditions and social pressures may give rise to those societal values. An objective outsider position within the data was adopted as none of the team members had experienced GDM.

### Ethical approval

Ethical approvals were sought and granted from King’s College London Research Ethics Committee (reference numbers: HR/DP-21/22-26417 and LRS/DP-22/23-34503).

### Patient and public involvement

A patient and public involvement (PPI) group is involved with this research. All women (*n* = 5) in the group have experienced GDM and have provided feedback on the research question, study documents, interview schedules, as well as recruitment strategies.

### Recruitment and participants

Women were recruited through advertisements in diabetes-related media and social media as well as pregnancy support groups. Eligibility criteria included age (18 years or older), country of residence (UK only), GDM diagnosis (current or within the past 4 years) and language (ability to communicate in English). Interested potential participants responded to the advert via the research team’s e-mail address. Prospective participants were provided the participant information sheet and consent form and a date and time suitable for women was arranged for interviews.

The *n* = 53 women were interviewed between January 2022 and February 2023. They were aged between 23 and 43 years (mean = 34 years), *n* = 13 women were in the antenatal period and *n* = 40 women in the postnatal period at the time of their interview. *N* = 4 women had a parity of 0, *n* = 22 a parity of 1, *n* = 21 a parity of 2, and *n* = 6 a parity of 3 or more. *N* = 36 women had one GDM pregnancy, *n* = 14 had experienced two GDM pregnancies, and *n* = 3 women had experienced three or more GDM pregnancies. See Supplemental Appendix 1 for ethnicity of participants.

### Data collection

Interviews were conducted primarily by one researcher (MB), remotely using video-conferencing software. A slightly different interview schedule was used for each study, but consent to participate and demographic data were collected at the beginning of every interview. The main focus of the initial study was on psychological outcomes and mother-infant bonding, whereas the extended, second study focused on women’s ‘social experiences’, whilst indirectly questioning around their experiences of stigma, but not explicitly referring to it. In both cases, participants were invited to discuss their own experiences in a range of contexts, including healthcare settings, the workplace, their social and/or family environments and in the media. The word ‘stigma’ was not used until either the participant had stated it spontaneously or until the last question was asked to address this concept directly. This approach was used to avoid confusing participants with jargon, and to avoid introducing bias to questioning, thus maximising opportunities for participants to discuss their positive and negative social experiences. The semi-structured nature of interviews (McIntosh and Morse, 2015) allowed flexibility for the researcher to pursue interesting lines of inquiry pertinent to and raised by individual participants. Interviews ranged between 25 and 69 minutes in length (mean = 40 minutes) and were recorded and transcribed verbatim by either the research team or a professional transcriber. Transcripts were read (while listening to original audio) and re-read by the lead researcher (MB) to check for accuracy.

### Data analysis

Grounded Theory is a rigorous and methodical qualitative research methodology, which aims to generate a theoretical explanation for a phenomenon of interest from participants’ narratives (Holton and Walsh, 2016). Grounded Theory (Glaser and Strauss, 1967) is therefore a well-suited analytic technique to explore a specific population’s (e.g. women’s) experiences of a specific phenomenon (e.g. stigma), in a specific context (e.g. when diagnosed with GDM), and thereafter contribute new theory to the literature-base. Grounded Theory follows an inductive, data-driven, approach to analysing interview transcripts (Holton and Walsh, 2016). In the present study, transcripts were manually coded using open, line-by-line coding, then re-read and focus coded by two researchers (MB, NH) independently, and then cross-checked and discussed collaboratively. Memo writing during coding was reflected upon during the first stages of theory generation. Following guidance (Silverio et al., 2019) researchers (MB, SAS, NH) met to discuss higher-order (focused) codes, super-categories, and the final theory generation. Theoretical saturation (Vasileiou et al., 2018) was understood to be achieved when data driven themes were adequately supported with quotations to enable theory formation.

**Figure 1.**
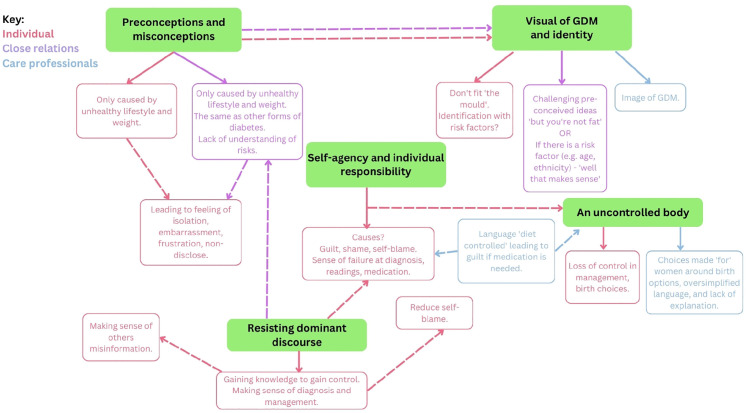
Thematic diagram of super-categories.

**Figure 2.**
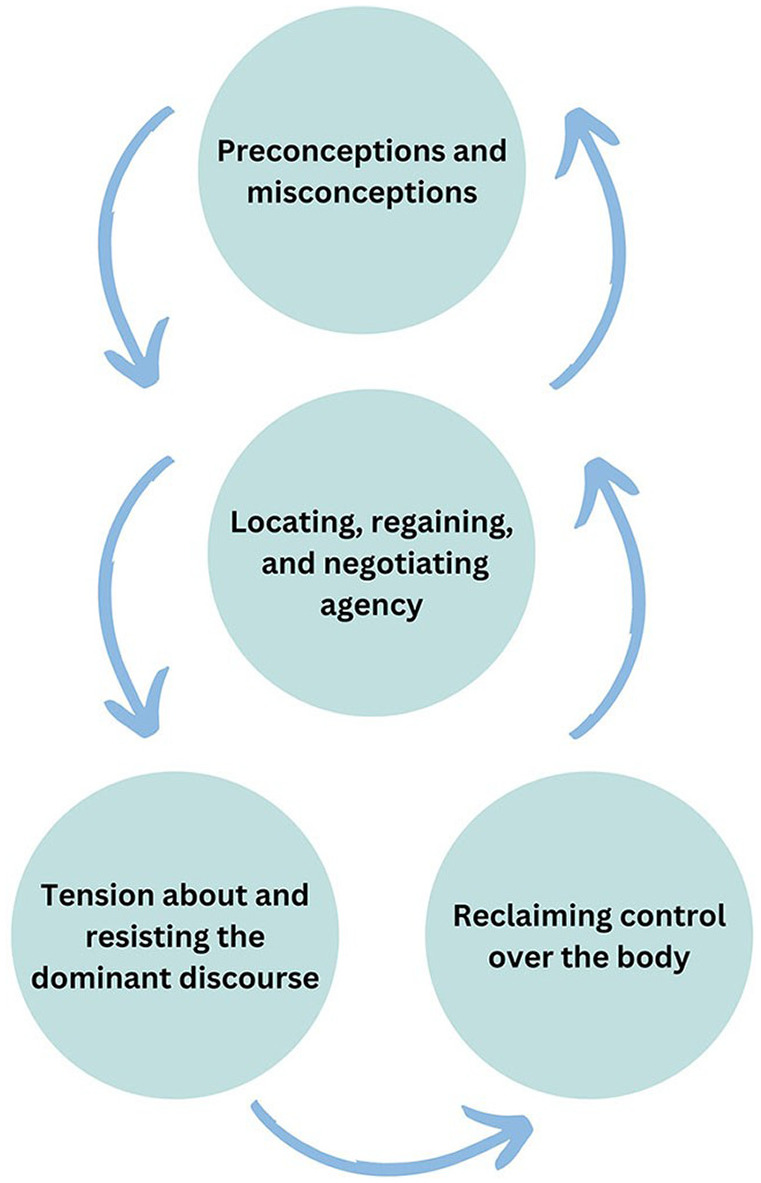
Thematic diagram of final themes in theory.

Thematic diagrams (Silverio et al., 2019) were used to facilitate theory generation and finalise theory formation (Figure 1 illustrates the thematic map of the super-categories and Figure 2 illustrates the final theory). In finalising the Grounded Theory, the research team discussed, challenged and provided defence of the theory to establish a coherent narrative of the data, outlining the processes and mechanisms accounting for the patterns observed between themes, as shown in the thematic diagram.

## Results

The analysis yielded four primary themes: (1) Preconceptions and misconceptions; (2) Locating, regaining, and negotiating agency; (3) Tension about and resisting the dominant discourse of stigma; and (4) Reclaiming control over the body. These were generated from a series of super-categories (see Figure 1), which themselves were derived from line-by-line and focused coding. Themes are supported with illustrative quotations from the dataset. The final theory is illustrated by the interaction between the four themes (Figure 2).

### Preconceptions and misconceptions of GDM

Most women reported limited understanding and knowledge regarding GDM prior to diagnosis. Several women recounted their own preconceived notions and assumptions pertaining to the condition, such as the misconception that it exclusively affects individuals who are overweight or led an unhealthy lifestyle.

*Naively, I thought that I wouldn't have it cause I was a normal weight … fit and healthy … I hadn't even really entertained the idea that I'll get it.* (P:12)

Women discussed a ‘visual of GDM’ which was held by them and others around them. Some women described not fitting into a ‘mould’ of GDM. When they didn’t fit the perceived visual of GDM, described as being overweight or unhealthy, it led to enhanced feelings of shock and disbelief at diagnosis.

*they [family] were a bit shocked because I don't really sort of fit the stereotypical mould of the diabetic mum* (P:38).

*it was more of an initial shock more than anything . . . I know like there isn't an ideal look for . . . GDM, but I'm really fit and healthy. So I was really shocked.* (P:32)

Some women recounted instances where healthcare professionals made remarks regarding the perceived ‘physical’ appearance of GDM.

*This is my second pregnancy [that] I've had her as my consultant and she still says to me ‘you really don't look like somebody with GDM' I was like yeah, surprise!* (P:28)

Women discussed remarks and comments from family and friends surrounding their diagnosis which brought to the fore the existence of preconceived notions and misconceptions harboured by others. Preconceptions primarily pertained to the perceived risk factors for GDM, which subsequently became the subject of critical scrutiny and questioning.

*everyone was like, it's an overweight people's problem. And then when I did get it, they were like, ‘oh my god, she's not overweight and she's got GDM' and it kind of baffled people.* (P:43)

…*throw away comments ‘but you're not a big person' or ‘you're not an unhealthy person' or ‘ohh that's surprising that you've got it'. Which I guess that comment is being made because there's an assumption that maybe people who were larger or less healthy or did less activity were the sort of person that was going to get it.* (P:42)

Women often felt that those around them did not appreciate the impact of GDM including its intensive management and risk to baby. Furthermore, women recounted instances of family members drawing parallels between their own type 1 and 2 diabetes and that similar management strategies could be employed by women.

*I tried to explain to her [family member] the risk of stillbirth and having impacts on your own body afterwards and it evolving into type 2 and I don’t think she really got it, but I think people thought ‘Oh, well you’ll just have a big bouncing baby’.* (P:38)

*they didn’t realise the seriousness of it. I think they maybe thought it was something that I was exaggerating…the worst complications of it can be infant death. It’s awful.* (P:40)

Women’s preconceptions about GDM, along with family comments and sometimes a subsequent desire to avoid drawing attention to the condition, led to feelings of shame and hesitancy in disclosing their diagnosis to others. Women feared being blamed for the condition or perceived as causing something potentially harmful to their baby. Women felt frustration when close relations did not appreciate the demanding nature of the condition, leading some women to avoid social situations, which resulted in feelings of isolation.

*I haven’t really told anybody about it. I haven’t told family, I haven’t told my parents, I haven’t told my partner’s family because of the stigma that comes with being labelled as someone with gestational diabetes. Like a lot of them probably wouldn’t understand it and will probably have similar preconceptions to what I had in terms of… ‘it’s what happens to people who are unhealthy or people who haven’t been taking care of themselves’…I fear…the repercussions of people feeling like that…I think that’s probably impacted me more than actually having it.* (P:9)

*Initially I was actually embarrassed to tell people because again, I presumed that people would think that it was something that I had done.* (P:37)

Some women highlighted the term diabetes was associated with an assumption that the condition results only from lifestyle choices rather than other external and uncontrollable factors.

*the word diabetes, has this kind of lifestyle factor association to it and it’s sort of almost like oh so how do I tell people that I’ve got this disease that people naturally associate with being…unhealthy?* (P:15)

### Locating, regaining, and negotiating agency

Women discussed feelings of responsibility for their diagnosis which resulted in negative psychological responses including self-blame, shame and guilt and considerable worry for their baby’s short and long-term health.

*I’ve done something that’s going to harm my baby, it’s my fault, quite a lot of self-blame.* (P:8)

*I just felt very guilty because obviously that now puts my child at higher risk of developing type 2 and just like the fear of having that high blood sugar and thinking, oh my gosh, what is it doing to my baby.* (P:26)

Women discussed reflecting on preconception behaviours, lifestyles and foods they had eaten in pregnancy when trying to understand a cause for the diagnosis.

*Then there’s a lot of like guilt… part of you that just thinks did I eat too much chocolate…did I not do enough exercise? … I’m a bit older…is this somehow my fault? That guilt comes in and also…shame.* (P:15)

Women’s acceptance of a GDM diagnosis was influenced by their risk factors. Women from certain ethnic backgrounds, at higher risk of GDM, reported a normalised perception of the condition within their communities. Consequently, they described reduced stigmatisation and a greater level of acceptance upon receiving the diagnosis.

…*people speak about it… they just accept it as it’s very normal, we’re Bangladeshi, it’s going to happen, and it’s the same thing with… preeclampsia… I feel like in our community it’s very much an accepted thing…it’s just very natural. Like if you see someone, they’ve got GDM…it’s not looked down upon, it’s not frowned upon, it’s not something that anyone blames anyone for…almost every other person I know has had it in their pregnancy.* (P:41)

Women discussed feelings of failure in their experience with GDM. Women discussed *failing* the GDM screening test and subsequent blood glucose monitoring. Several women expressed a sentiment of physical inadequacy, feeling that their body had failed them. Additionally, some women felt they had failed their child and an inability to be an effective mother.

*I felt like I had failed the baby and I felt like I had failed the pregnancy and I felt like my partner was relying on me to carry his baby and we were gonna have a perfect family. And then all of a sudden, once I got diagnosed… it was like oh, I’ve not done something right…before being diagnosed I was eating a lot of quavers and I was having a lot of cravings and I just instantly put the blame on myself like ‘ohh, I shouldn’t have done that’.* (P:48)

Individual agency related to management was a common topic among women. Some women expressed a sense of loosing agency while trying to attain desirable blood glucose levels, and for some upon initiating pharmacotherapy to manage the condition.

*That (insulin therapy) felt like another like failure point that I couldn’t do something else to fix it just myself.* (P:15)

Women described experiencing a lack of autonomy while engaging in dialogues and decision making regarding their birth. Many women recounted instances where they received directives on the expected course of their labour, without being able to input themselves.

*a woman is still… able to make choices about…honouring that process that she’s going through and not using language like ‘we will not let you’…‘you won’t be able to go’, ‘we won’t let you pass to this number of weeks’, ‘we will induce you…’. It’s actually just making the assumption that, you know, you will be told what to do, you’re infantilised.* (P:1)

*in the world of having babies, it is generally very much like no one wants to tell you what to do…your choices and your options and…then… it went like bam the other way… it was very much… you have to do this and you have to do that. And I was like ‘but why?’. And didn’t feel like I was given…an explanation. And didn’t feel like I was given…an explanation.* (P:42)

Women discussed feeling like their bodies were thought of by HCPs as something women couldn’t control due to GDM and that HCPs had to take control to ensure the safe arrival of their baby.

*And then they layer that on top with like conversations about risk…some of the statements that I’ve heard are ‘the last thing I need is a stillborn on my hands’.* (P:1)

*The consultant appointments I found a bit patronising…‘We won’t let you do this and we are going to make you do that and this is how it’s going to go’. Very much box ticking. ‘You’ve got this condition and therefore this is going to happen’. Not ‘Would you like to do this?’ or, ‘These are your options’, or ‘Have you thought about that?’.* (P:49)

Some women feared taking medication due to worries about its effect on the baby and birthing choices. Those needing medication for GDM felt internalised stigma, leading to further feelings of inadequacy and failure. Women also described that the need for insulin therapies or metformin led to choice and control being taken away from them.

*The thing…I found hardest was the thought of going on medication. I don’t know why like, I know that there’s nothing wrong with going on medication and people need it and people have to do it, but it kind of felt like for me like a bit of a, like I’ve been defeated.* (P:21)

*it felt out of control…if I do get a high reading, that’s just the hill…that we’re starting to go down and it’s only going to start getting worse and more complex, more choices will be taken away from me and…baby is gonna…suffer more as a result.* (P:14)

Some women discussed frustration surrounding the term ‘diet controlled’ when managing their GDM and that it emphasised that GDM is something that can be ‘controlled’ by all women.

*I tend to get a lot of, ‘Oh well done that you’ve been able to stay diet controlled’ and in reality, yes, I’ve worked hard to be able to stay diet controlled, but sometimes people do that, and their body just doesn’t respond…so, it’s not only about what the mother is putting in, it’s a lot also how your body is responding to it, and I’ve just been lucky that my body responded to it.* (P:14)

### Tension about and resisting the dominant discourse of stigma

Women were more often than not faced with negative discourses by those around them, leading to pervasive feelings of shame about the condition.

*For me, personally, it’s probably all built around the whole fat shaming thing and the way that people treat you when you are overweight and they treat you like you are stupid and it’s your fault and it’s all completely within your control that you could just go on a diet and you could just be thin, but that isn’t actually the case…there’s a lot more to being overweight than what people give credit for and there are a lot of people who do just think you can go on a diet and you can be thin and that’s it, and that isn’t the case.* (P:49)

Women highlighted ways they tried to negotiate and resist dominant discourses to produce counter knowledge that offered new (and less oppressive) meanings and possibilities for being. This occurred throughout the perinatal period and included tackling misinformation about GDM directly.

*I also think that the stigma specifically with diabetes when you get out the needles to do the test and stuff, people are sort of looking at you like are you injecting heroin or something. It’s like if I was injecting heroin, I wouldn’t be doing it blatantly in the middle of a shop or a restaurant…I specifically try not to hide when I do it because I think well no I’m not going to because why should I?* (P:49)

Many women described coming to terms with other people’s stigmatising views surrounding GDM as coming from a place of ignorance.

*It’s more of an ignorant thing. I think people think they’re being funny, but they’re not. It’s not funny. It’s more a lack of understanding on their part and it’s frustrating.* (P:40)

#### Reclaiming control over the body

When discourses were internalised, this led to the sense of lack of control over women’s pregnancies, especially for those whose pregnancies became increasingly monitored and medicalised.

*I’ve now got health anxiety and I’m having to deal with it…now I feel like health is like a control thing… I was like, you’ve got something wrong… it’s usually like if you’re overweight, just lose some weight or you’ve got bad breathing and you smoke a lot. Stop smoking… it’s always told that you do something to better yourself or make yourself better or go out and do a bit more walking or…I put everything into it [GDM] and I know the food I ate was healthy and good and it didn’t work. So, I felt that lack of control and then obviously being induced and that failed. I felt like, well, I’ve lost control of that as well.* (P:43)

Many women discussed engaging in GDM support communities online which increased their understanding of the condition and reduced self-blame, shame and feeling isolated. Women recounting that developing a deeper understanding of the condition meant they could understand that factors outside their control could have caused the condition.

*…talking to other people, you know, helps, particularly people that have already been in that position. It starts to make you feel like okay you’re not alone. This really is quite a common thing…it’s not something that necessarily I’ve done wrong.* (P:15)

*Women not only discussed the necessity of educating family, friends, and others about GDM but also described the process of coming to terms with the stigmatising views held by others, acknowledging that these views often stemmed from a place of unawareness. “It’s more of an ignorant thing. It’s more a lack of understanding on their part and it’s frustrating…they’d be like, ‘Oh but you’re small, how can you have it?’ …then you can kind of prep people and go, ‘Oh it can happen to anybody, any shape or size, it doesn’t matter’”*. (P:40)

## Discussion

In summary, this grounded theory analysis revealed that women’s experiences with GDM were shaped by preconceived notions and misconceptions, leading to feelings of shame, guilt, and isolation. Women sought to regain agency by challenging dominant views and regaining control over both their pregnancy and condition, and also the societal misconceptions and narratives which were extant and incorrectly communicating what GDM was, its origins, and course. Tension and resistance highlighted the complex interplay between intrapersonal, interpersonal and broader societal stigma, and women’s efforts to reclaim control over their health and well-being during the GDM journey. Our theory – the (un)controlled body – of the experiences of stigma by women with GDM, demonstrated that over the course of a pregnancy many women experience stigma from varied sources related to different aspects of the condition. Women describe actively resisting stigma, first by learning themselves about GDM, and then by tackling dominant discourses often perpetuated by those around them – including friends, family and on occasion healthcare professionals.

Overall, women’s experiences of GDM-specific stigma were diverse and far-reaching. Stigma was experienced in numerous social settings, from healthcare professionals, within the media, interpersonal network (family) and also internalised. We highlight the intersectionality and convergence of other stigmatised conditions within GDM-specific stigma namely; weight stigma in preconception, pregnancy and postpartum (Hill and Incollingo Rodriguez, 2020); and with more broader diabetes-related stigma (Akyirem et al., 2023). We demonstrate compounding experiences of stigma where those experiences are amplified when women face both weight- and GDM-related stigma. Previous research highlights the intersectionality of stigma is a key contributor to health inequalities, given its complex and multifaceted consequences (Hatzenbuehler et al., 2013). Although the consequences of GDM-specific stigma are relatively unexplored, if we draw on the knowledge from other stigmatised conditions, the negative outcomes are concerning and underscore the importance of addressing GDM-specific stigma. We also highlighted that stigma experienced by women with GDM is particularly unique given the context of pregnancy, specific weight gain expectations and social norms surrounding pregnancy (e.g. maternal health behaviour expectations and pregnant body ideals, as well as social ideals for women to ‘protect’ their baby).

Limited understanding and knowledge regarding GDM among women prior to diagnosis was highlighted which was closely linked to preconceptions and misconceptions about the condition, for example, that GDM exclusively affects individuals who are overweight or lead an ‘unhealthy’ lifestyle. This was not only experienced at an individual level but also from close relatives, friends and at a broader societal level. Stigma from family and friends was highlighted including misconceptions around the conditions, negative comments that assume the poor health of the pregnant woman, projecting lifestyle and behavioural stereotypes with GDM (i.e. poor diet and physical inactivity), women also experienced increased scrutiny from other around them in relation to the management of GDM. Observed oversimplification of the causes of GDM ignored the complex and inter-related physical, environmental and social causal factors, many of which fall outside of individual control. These findings are echoed in previous literature, which have highlighted that the most frequently experienced stigma related to type 1 diabetes and T2D, was the perception that the diagnosis was a result of having a character flaw or due to failure of personal responsibility (Liu et al., 2017). In the context of pregnancy, recent literature highlights that regardless of BMI or obesity diagnosis, pregnant women experienced weight-stigmatising comments from close relations, including partners, family and friends (Nagpal et al., 2023). Weight-stigmatising experiences in these relationships included making negative assumptions about maternal health and foetal development based on gestational weight gain, regardless of whether that gain is perceived as too much or too little. It has further been noted that weight-stigmatising comments project traditional stereotypes associated with weight gain, namely that it stems from poor lifestyle behaviours (Nagpal et al., 2023). Women reported that the misunderstanding of the condition by those around them impacted on social support. Given that close relations can be integral sources of social support during pregnancy and postpartum, advocacy for prevention of GDM-specific stigma should extend to close networks so they can serve as a protective factor for maternal health outcomes. Women also described remarks from healthcare professionals about the physical appearance of GDM, thereby perpetuating the stereotype that only specific individuals are at risk of the condition.

Women often began by locating agency within themselves, leading to feeling a sense of responsibility for diagnosis. This internalisation of responsibility resulted in negative psychological responses, including self-blame, shame, guilt and worry for the baby’s health. The burden of this responsibility, combined with the perceived lack of understanding and support from others, further contributed to women’s reluctance to disclose their condition. Self-agency was also impacted on by language used by healthcare professionals. Previous research has highlighted the potential problematic use of the phrase ‘diet-controlled’ in GDM management which may be contributing to increased stigma faced by women with GDM, as when the GDM is not manageable through diet, feelings of failure, shame and self-blame can arise (Elton, 2022). Women in this study expressed concerns relating to taking medication for GDM and how this may impact their pregnancy and baby’s health. Similar qualitative studies have reported that women experience feelings of responsibility and guilt related to their unborn child, and feeling like a failure if they need to resort to medication for management (Draffin et al., 2016; Parsons et al., 2018). Women’s acceptance and understanding of their diagnosis was influenced by identified risk factors. For instance, women from certain ethnic backgrounds, at higher risk of developing GDM, reported a normalised perception of the condition within communities. Consequently, these women reported reduced stigmatisation of the condition from outside sources. This is contrary to a previous review, highlighting that women experienced direct GDM discrimination from family members in some ethnic communities (Davidsen et al., 2022). Further, in other literature, for some women in India and China, and in Native American populations, they were blamed by their mother-in-laws for carrying ‘unhealthy babies’ and keeping their diabetes a secret before marriage (Ge et al., 2017; Nielsen et al., 2012; Stotz et al., 2019). Women discussed a lack of autonomy in decision-making regarding their management and birth choices, intensifying feelings of inadequacy and loss of control. This is consistent with contemporary thinking on stigmatisation, that the root cause of its effect on people is because the discourse being shared removes agency from the person being stigmatised; which in a highly medicalised and monitored pregnancy as those experienced by women with GDM, can add to the feeling of not being in control of their bodies, or certainly their short-term futures.

Women’s experiences of stigma changed over time. This is demonstrated in that women initially discussed not having knowledge about the condition and then blaming themselves, to gaining knowledge, reduced self-blame, shifting agency, and for some then educating others. Previous research has highlighted the importance of accounting for time in stigma research which can yield key insights into how experiences of stigma evolve, how pathways link stigma with health change and when individuals are most vulnerable or resilient to the effects of stigma (Earnshaw et al., 2022). Considering time and the different points in time women with GDM may experience stigma, highlights the importance of identifying appropriate intervention targets for specific times and considering which stigma reduction tools would be most effective at which time points (Earnshaw et al., 2022). Women describe efforts to challenge and counter the dominant views surrounding GDM. Engaging in peer support communities on social media helped women gain a deeper understanding of the condition, reducing self-blame, shame and isolation. Educating others about GDM and coming to terms with others’ stigmatising views were strategies employed by women to resist the prevailing misconceptions and regain control over their experiences. However, the burden of having to educate or counter stereotypes and stigmatising views of those around them was a significant on women especially when they already felt considerable responsibility was placed on them in terms of management. Previous research has highlighted than women with GDM report a lack of control at initial diagnosis, which over time women transitioned from feeling like a victim of diabetes, to being active agents in controlling their GDM (Craig et al., 2020). By gaining knowledge about GDM and investigating alternative options to management such as outside those recommended by HCPs, provided women with some autonomy in managing their condition (Craig et al., 2020).

### Implications

Theoretical models and frameworks have been developed to investigate pregnancy-related weight stigma and its consequences (Incollingo Rodriguez and Nagpal, 2021). Future studies should examine the overlap of GDM and weight stigma to support stigma reduction initiatives for pregnant and postpartum women. The impact of GDM-specific stigma on clinical outcomes has not been examined. However, if we draw parallels from literature on weight stigma in preconception, pregnancy and the postnatal period, and on stigma in diabetes, the consequence of GDM-specific stigma are concerning. Weight stigma has been associated with low self-efficacy for engaging in physical activity and increased risk for disordered eating (Lessard et al., 2021; Pearl et al., 2017). Meta-analytic evidence demonstrates a moderate and reliable relationship between weight stigma and poor mental health indicators (e.g. depression, anxiety, distress) (Emmer et al., 2020) and poor physical and mental health outcomes which can exacerbate weight-related comorbidities (Puhl and Suh, 2015). Furthermore, recent research has also linked experienced weight stigma in pregnancy with an increased risk of developing GDM, and that this relationship was stronger than the relationship between BMI and GDM (Nagpal et al., 2021). Women diagnosed with GDM who experience stigma, may face more barriers to engage in healthy behaviours than women who do not experience stigma.

We identified several short-term consequences of GDM-specific stigma including self-blame, guilt, shame, anxiety, isolation, and impact on pregnancy decisions, which can indicate a potential high risk of long-term consequences for women who experience GDM-specific stigma. This study emphasises the need for increased awareness and education about GDM to challenge preconceived notions and misconceptions surrounding the condition. Interventions aimed at reducing stigma and providing social support to women with GDM should be developed. Creating safe spaces for women to share their experiences, concerns and challenges can help alleviate feelings of isolation and promote emotional well-being. Peer support programmes, online forums and educational resources can play a crucial role in empowering women and challenging societal misconceptions surrounding GDM.

### Strengths, limitations, and future research

We recruited women from both the antenatal and postnatal period which assisted in understanding how perceptions and psychosocial implications of GDM can change over time, however, it would be useful to conduct a longitudinal qualitative study with a sample of consecutive women. Online sampling may have led to a sample of women skewed towards having higher education and socioeconomic levels, and being from primarily white ethnic groups (Topolovec-Vranic and Natarajan, 2016). Cultural and contextual factors specific to the UK may have influenced participants’ experiences of stigma. Women’s experiences may differ in countries with different healthcare systems and approaches to diagnosis and management of the condition, different societal perceptions of the condition and women’s health and bodies more generally. We also acknowledge that some women in our study experienced their GDM pregnancy during COVID-19, when they would have experienced unprecedented changes to their lives and more specifically maternity care. Future work should consider developing validated assessment tools to estimate the prevalence and screen for GDM-specific stigma, which will allow investigation of other factors that may moderate GDM-stigma experiences. It is also important to understand how GDM-specific stigma impacts on the risk of long-term health conditions including T2D.

## Conclusion

Our study underscores the multifaceted nature of stigma surrounding GDM and emphasising its convergence with weight stigma during pregnancy and the need for targeted stigma reduction initiatives and validated assessment tools.

## Supplemental Material

sj-docx-1-hpq-10.1177_13591053241241863 – Supplemental material for The (un)controlled body: A grounded theory analysis to conceptualise stigma for women with gestational diabetes mellitus Supplemental material, sj-docx-1-hpq-10.1177_13591053241241863 for The (un)controlled body: A grounded theory analysis to conceptualise stigma for women with gestational diabetes mellitus by Madeleine Benton, Natasha Hotung, Jessica Bird, Khalida Ismail and Sergio A Silverio in Journal of Health Psychology
