# Supplementary material for: The (un)controlled body: A grounded theory analysis to conceptualise stigma for women with gestational diabetes mellitus
Source: J Health Psychol. 2024 Apr 16;30(5):871–86. doi: 10.1177/13591053241241863 (PMC11977814; doi:10.1177/13591053241241863)
Supplement: sj-docx-1-hpq-10.1177_13591053241241863 – Supplemental material for The (un)controlled body: A grounded theory analysis to conceptualise stigma for women with gestational diabetes mellitus [file sj-docx-1-hpq-10.1177_13591053241241863.docx]

**Appendix 1 – Table 1.** Ethnicity of n=53 participants.

| **Participant Ethnicity*** | **N=53 (%)** |
| --- | --- |
| White British | 32 (60%) |
| White Other | 7 (13%) |
| Mixed Asian | 2 (4%) |
| African | 1 (2%) |
| Bangladeshi | 1 (2%) |
| Black Caribbean | 2 (4%) |
| Black African | 1 (2%) |
| Chinese | 1 (2%) |
| Indian | 2 (4%) |
| Japanese | 1 (2%) |
| Pakistani | 2 (4%) |
| Sri Lankan | 1 (2%) |

Note: Ethnicity was defined by participants in response to the question: “Could you tell me the ethnicity with which you identify?”
